# A case of diffusely infiltrating colorectal cancer with distinctive endoscopic and histologic findings

**DOI:** 10.1016/j.igie.2022.12.002

**Published:** 2023-02-08

**Authors:** Takashi Obana, Katsuji Tokuhara, Hirohiko Miyake, Yasuhiro Sakai, Eri Shimada, Mitsuo Kishimoto

**Affiliations:** 1 Department of Gastroenterology, Komatsu Hospital, Osaka, Japan; 2 Department of Gastroenterological Surgery, Kansai Medical University Medical Center, Osaka, Japan; 3 Department of Diagnostic Pathology, Kansai Medical University Medical Center, Osaka, Japan; 4 Department of Surgical Pathology, Kyoto City Hospital, Kyoto, Japan

A man in his 50s visited our hospital for positive fecal immunochemical tests and transient abdominal pain. Colonoscopy revealed multiple diverticula and localized reddish, granular mucosa with mildly converging folds at the ascending colon (Fig. 1). Narrow-band imaging magnifying endoscopy visualized normal pits (Kudo’s type I pit pattern) on the reddish mucosa (Fig. 2). Endoscopically, the findings were not suggestive of neoplastic changes. Malignancy was not proven by biopsy samples.

**Figure 1:**
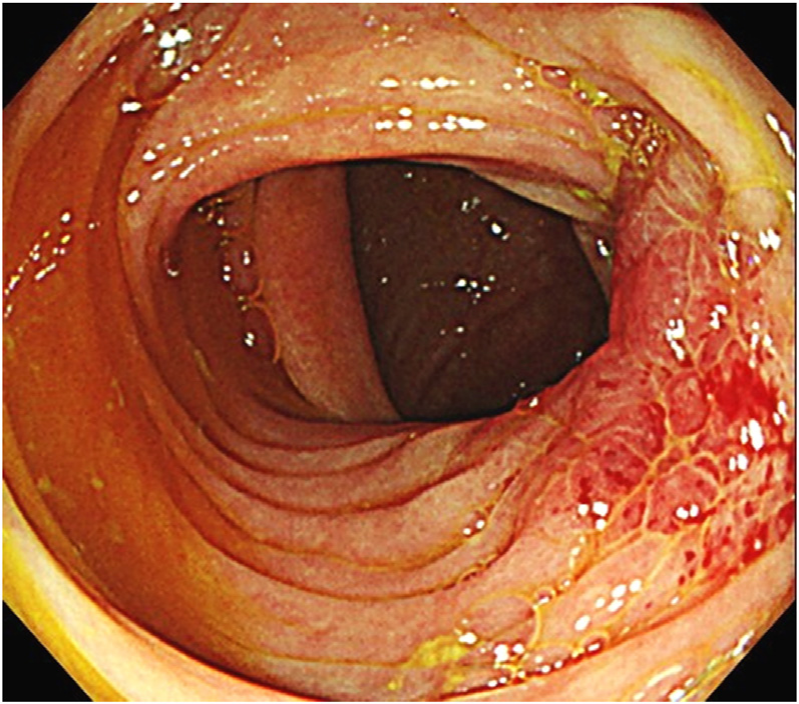
Colonoscopy showed localized reddish, granular mucosa with mildly converging folds at the ascending colon.

**Figure 2:**
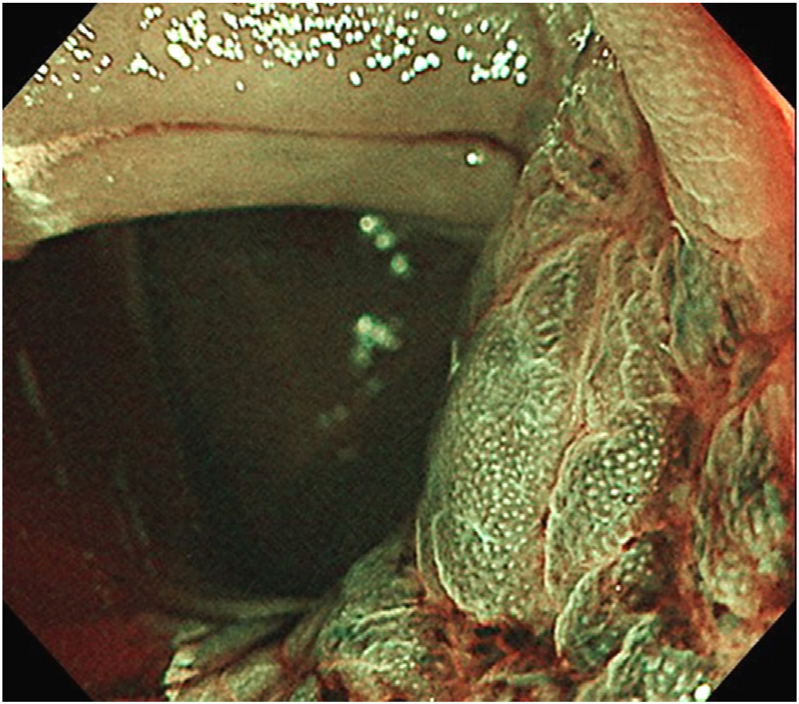
Narrow-band imaging magnifying endoscopy visualized normal pits on the reddish mucosa.

One year later, he underwent colonoscopy again after another positive fecal immunochemical test. The ascending colon became severely strictured because of worsening converging folds. An enlargement was seen in the reddish mucosa, but ulcerations were absent (Fig. 3). Adenocarcinoma cells were detected in the biopsy specimens. Other malignant neoplasms were not revealed in the chest and abdomen by EGD and contrast-enhanced CT; thus, the lesion was considered to be primary type 4 (the Paris endoscopic classification) ascending colon cancer. The patient was transferred to a tertiary referral institution.

**Figure 3:**
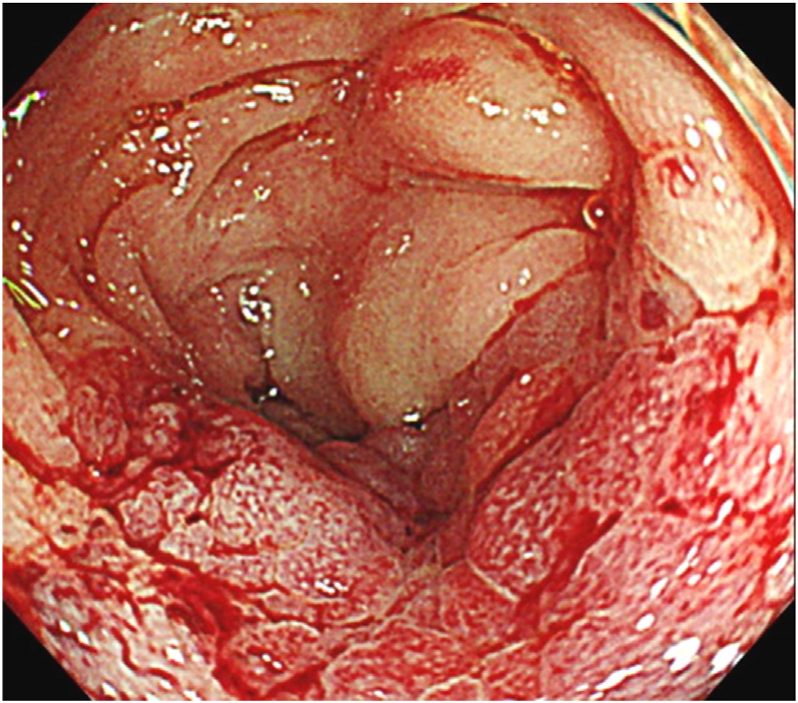
On second colonoscopy, the area of reddish mucosa enlarged, and the lumen became severely stenosed due to worsening converging folds.

Although the preoperative TNM stage was cT3N0M1c cStage IVc, intraoperative peritoneal washing cytology was negative. Therefore, a right hemicolectomy was performed. The resected tumor was 31 × 20 mm in size. Histologically, the diffuse spread of well to moderately differentiated adenocarcinoma cells were observed, accompanied by significant desmoplastic reactions (Figs. 4 and 5). Although the tumor partially infiltrated into the subserosal layer, most of the mucosal surface was covered with normal glands, having only focal cancer exposure (Fig. 6). The patient was finally diagnosed with diffusely infiltrating ascending colon cancer with predominantly differentiated histology.

**Figure 4:**
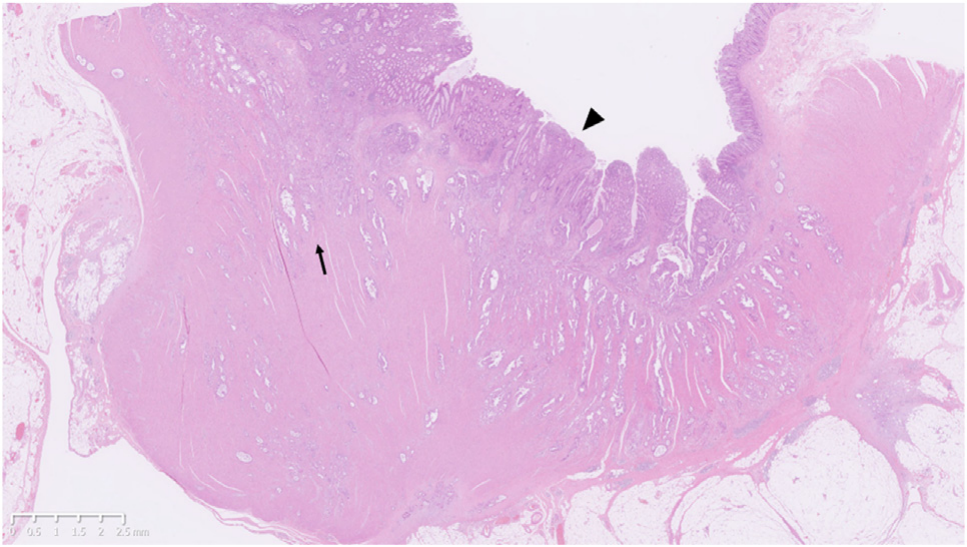
In the resected specimen, diffuse spread of well to moderately differentiated adenocarcinoma cells was observed with marked stromal desmoplastic reactions (H&E, orig. mag. ×30).

**Figure 5:**
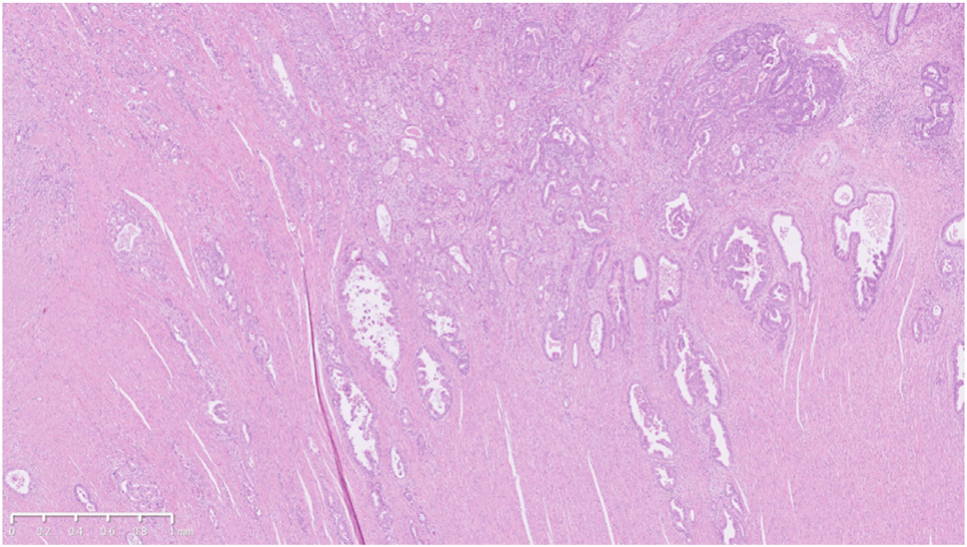
The higher-power field image of arrow on Figure 4 (H&E, orig. mag. ×30).

**Figure 6:**
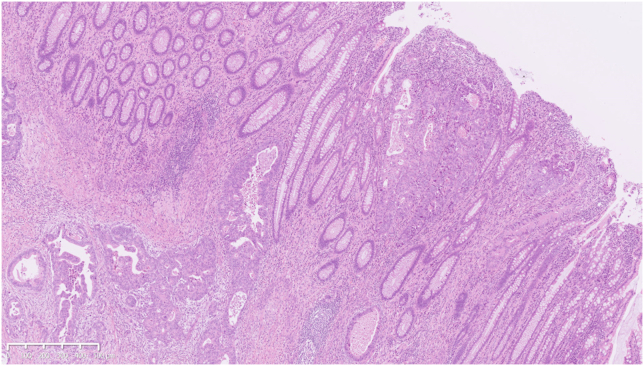
Most of the mucosal surface was covered with normal glands. Carcinoma cells were only focally exposed (arrowhead on Figure 4; H&E, orig. mag. ×50).

The initial endoscopic findings seemed to reflect those of diffusely infiltrating colorectal cancer at a relatively early stage and should be noted. The chronological changes mentioned above also shed some light on the growth pattern of this particular disease. This case was considered to be distinctive from the perspective of endoscopic and histologic features.

## Patient Consent

The authors have received appropriate patient consent for the publication of this article.

## Disclosure

*All authors disclosed no financial relationships.*

